# Paternal germ line aging: DNA methylation age prediction from human sperm

**DOI:** 10.1186/s12864-018-5153-4

**Published:** 2018-10-22

**Authors:** Timothy G. Jenkins, Kenneth I. Aston, Bradley Cairns, Andrew Smith, Douglas T. Carrell

**Affiliations:** 1 Andrology and IVF Laboratories, University of Utah, 675 Arapeen Dr. Suite 201, Salt Lake City, UT 84108 USA; 2 Huntsman Cancer Institute, Salt Lake City, USA; 3 University of Southern California, Los Angeles, USA; 4 Department of Obstetrics and Gynecology, University of Utah, Salt Lake City, USA; 5 Department of Genetics, University of Utah, Salt Lake City, USA

**Keywords:** Sperm epigenetics, Aging, DNA methylation, Aging calculator

## Abstract

**Background:**

The relationship between aging and epigenetic profiles has been highlighted in many recent studies. Models using somatic cell methylomes to predict age have been successfully constructed. However, gamete aging is quite distinct and as such age prediction using sperm methylomes is ineffective with current techniques.

**Results:**

We have produced a model that utilizes human sperm DNA methylation signatures to predict chronological age by utilizing methylation array data from a total of 329 samples. The dataset used for model construction includes infertile patients, sperm donors, and individuals from the general population. Our model is capable predicting age with an R2 of 0.89, a mean absolute error (MAE) of 2.04 years, and a mean absolute percent error (MAPE) of 6.28% in our data set. We additionally investigated the reproducibility of prediction with our model in an independent cohort where 6 technical replicates of 10 individual samples were tested on different arrays. We found very similar age prediction accuracy (MAE = 2.37 years; MAPE = 7.05%) with a high degree of precision between replicates (standard deviation of only 0.877 years). Additionally, we found that smokers trended toward increased age profiles when compared to ‘never smokers’ though this pattern was only striking in a portion of the samples screened.

**Conclusions:**

The predictive model described herein was built to offer researchers the ability to assess “germ line age” by accessing sperm DNA methylation signatures at genomic regions affected by age. Our data suggest that this model can predict an individual’s chronological age with a high degree of accuracy regardless of fertility status and with a high degree of repeatability. Additionally, our data suggest that the aging process in sperm may be impacted by environmental factors, though this effect appears to be quite subtle and future work is needed to establish this relationship.

## Background

Recently, a great deal of work has been performed in an effort to understand the nature of aging, the mechanisms that drive the process, and the biomarkers that may be predictive of, or affected by, age. In this effort, a seminal manuscript was published in 2013 which described the ability to use DNA methylation signatures in somatic tissues to predict an individual’s chronological age [1]. In this work, Dr. Horvath demonstrated that the epigenetic mechanisms that reflect the aging process are tightly conserved between individual tissues and across multiple species. Remarkably, these patterns are sufficiently consistent to enable accurate age prediction with Horvath’s age calculator despite the significant contrast in epigenetic profiles between various somatic tissues.

Despite the general applicability of this model across diverse tissues, one tissue in particular did not display similar predictive power as was seen with most. In fact, DNA methylation signatures from testicular tissue and sperm specifically did not appear to be predictive of age at all with the previously described calculator [1]. In agreement with this observation is data from our lab which suggests that the nature of age associated alterations to sperm DNA methylation signatures are opposite of what is typically seen in somatic cells [1–4]. Specifically, although aging results in a global decrease in methylation and increased regional methylation in most cell types, we demonstrated that sperm exhibits the opposite trend. In many ways such a finding is not surprising as this is not the first case where the male germ line defied conventional age-associated cellular alterations. The most well described example of this is that of age impacts on telomere length. A hallmark of aging in somatic cells is a marked shortening of telomeres, but in sperm telomere lengthening is commonly seen with aging [5]. Clearly, sperm cells are extraordinarily unique and thus it seems likely that a unique approach is required to understand both the nature of the aging process and the potential predictive power of age associated alterations to the sperm epigenome.

In our previous publications we have described the general impact of aging on the sperm methylome. In these studies, we have shown that sperm have a very distinct pattern of age-associated alteration [2, 3]. We identified 148 genomic regions (~ 1 kb in size) that displayed differential methylation with age. Of these, only 8 displayed an increase in methylation, and the remaining 140 regions experienced a marked loss of methylation with age. Intriguingly, these regions of differential methylation are enriched at genes known to be associated with bipolar disorder and schizophrenia, both diseases known to have increased incidence in the offspring of older fathers. Indeed the epigenetic patterns of aging in sperm, while distinct from the epigenetic patterns of aging in somatic tissues, are striking and extremely consistent and thus provide an excellent opportunity for predictive model construction.

The pursuit of generating a model to predict an individual’s age using the sperm methylome is not only an interesting question from the perspective of basic cell biology but the patterns of sperm aging, and the unique nature of the sperm make the utilization of this cell type ideal for such a predictive model. Using pure cell populations is ideal for any epigenetic analysis, and while the previously constructed models are effective at predicting age even with tissues that are difficult to purify (which is a testament to quality of model and to the strength of the aging signal), the ideal scenario would be to use a pure cell population. Human sperm offer just such an opportunity. Many protocols are applied to somatic cell removal in sperm epigenetic studies and they have proven quite effective at isolating only germ cells, thanks in large part to the highly unique and compact nature of the sperm head. Further, the magnitude of the aging signal is quite strong in the sperm (thought to be in part due to the highly proliferative nature of the sperm cells themselves) and as a result, the patterns of aging offer an excellent opportunity for powerful prediction. In this study, we set out to capitalize on these advantages to build a model that can predict an individual’s age using methylation signatures in the paternal germ line. The experiments outlined herein describe the utility of the germ line age calculation and also provide evidence to suggest that the rate of aging can be affected by environmental exposures or lifestyles (smoking, obesity, etc.).

## Results

### Model construction and training

In the current study we assessed sperm DNA methylation array data (Illumina 450 K array) from 3 distinct previously performed studies [2, 6, 7]. From these data sets, we were able to utilize a total of 329 samples that were used to generate the predictive model outlined herein. Individuals with many different fertility phenotypes provided the samples used in this study. Specifically, our training data set includes samples from sperm donors [2], known fertile individuals, infertility patients (including those seeking intrauterine insemination or even in vitro fertilization treatment at our facility), and individuals from the general population [6, 7]. Further, our data set includes those that have very different lifestyles and environmental exposures (as an example, both heavy smokers and never smokers are represented in our data set).

We utilized the glmnet package in R to facilitate training and development of our linear regression age prediction model [8]. Beta-values were used in all experiments. These values represent fraction methylation as the standard output from the Illumina methylation array, which are scored between 0 and 1 with 0 representing complete demethlyation and 1 representing complete methylation. For training of our model, we first tested multiple designs to generate the most robust and easily interpretable model. We first constructed a model trained on all CpGs on the entire array (“entire array” training). We additionally limited the training dataset to only 148 regions that we have previously identified to be strongly associated with the aging process to ensure the broad interpretability to the results of the model [2]. We trained two models within those 148 genomic regions to identify the best possible outcomes. First, we trained on all of the beta-values for each CpG located in our regions of interest (“CpG level” training). Second, we generated a mean of beta-values for each region that included the CpGs within each region respectively yielding mean beta-values for each region (“regional level” training), and the model was trained only on these averages.

**Fig. 1:**
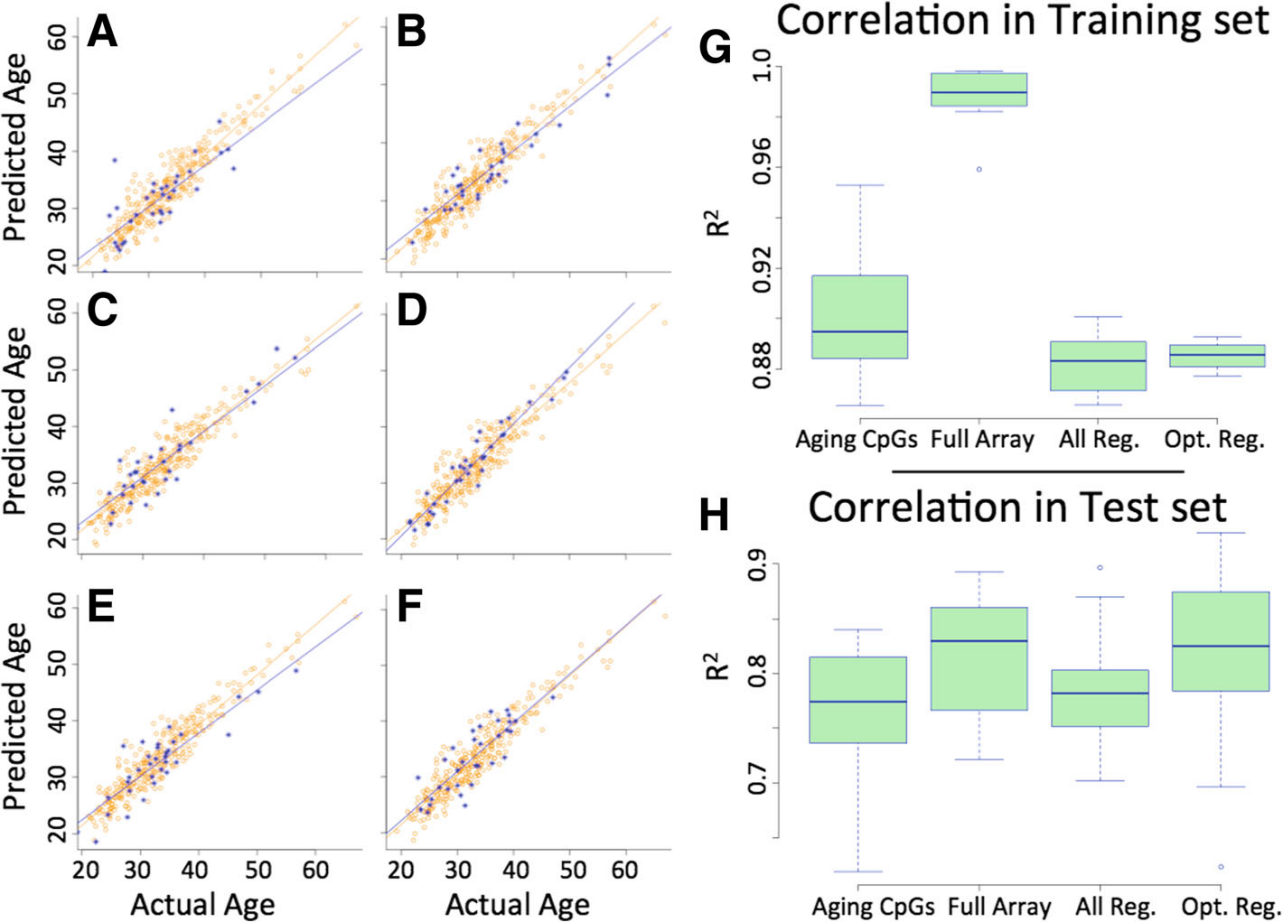
**a-f** Scatterplots depicting the relationship between predicted and chronological age in 6 represented models from our cross validation testing. **g** Box and whisker plots of the R2 values (predicted vs. actual) for the training data set from each cross validation for all four potential model designs including the CpG level training across the entire array and only those within the age-affected regions, as well as the full regional data set (148 regions) and the optimized regional data set (51 regions). **h** Box and whisker plots of the R2 values (predicted vs. actual) for the test data set from each cross validation for all four potential model designs including the CpG level training across the entire array and only those within the age-affected regions, as well as the full regional data set (148 regions) and the optimized regional data set (51 regions)

In each of the above-described scenarios, we employed a 10-fold cross validation strategy. This was performed 10 times on unique subgroups of the entire data set (Fig. 1a-f). The results from these ten validations were compared between the CpG level training and the regional level training. To compare the accuracy and predictive power of these models we performed linear regression for each (actual age vs. predicted age) and generated R^2^ values. These R^2^ values were compared via simple two-tailed t-test to determine if any significant differences exist between the various approaches to model construction (entire array, CpG level construction, regional level construction). These tests revealed that when only considering the prediction results on samples within the training set (those samples upon which the model was trained) there was a significant increase in predicative power when training on the entire array compared to any other approach (Fig. 1g) and that there was a modestly significant decrease in predictive power in the regional model (when compared to the CpG level model; *p* = 0.0428). However, when applying the predictive model to the test dataset these differences were no longer seen. In fact, there were no significant differences seen between the predictive capacities of the different models in these test sets (Fig. 1h). In an effort to make the model as simple as possible and in light of these findings, we committed to use the regional level model moving forward. Further, the alterations that occur at single CpGs are less likely to be biologically meaningful than those that occur over a region of the genome, thus using the regional level feature set improves the biological interpretability.

We additionally assessed the weighting of the features (regions) used in the models constructed during cross validation. We found a great deal of variation in the features selected across the regions screened, though a subset of the regions were heavily weighted and used in 80% or more of the models built during cross validation (a total of 51 features/regions met this criterion). In an effort to identify the simplest model we compared cross validation (10-fold strategy) in only these 51 regions (“optimized regions”) to all of the regions previously screened. We found that both the training and test groups were not statistically different between the optimized regional list and the full regional list (Fig. 1h). Further, the best performing model (and ultimately the selected model from our work) of any we tested was trained only on the optimized list of 51 regions of the genome (Table 1). In the training data set this model performed quite well with an r^2^ = 0.93, and similar predictive power was seen when screening all 329 samples in our data set (r^2^ = 0.89). To further highlight the power of prediction of this model it is helpful to note that our model predicted age with a mean absolute error (MAE) of 2.04 years, and a mean absolute percent error (MAPE) of 6.28% in our data set, thus the average accuracy in prediction is approximately 93.7%.

**Table 1:** Genomic regions used for age prediction

Name	CHR	Start	Stop
ADAMTS8	chr11	130,299,298	130,299,948
ARC	chr8	143,694,010	143,694,548
ARGHGEF10	chr8	1,877,888	1,878,324
BCL11A	chr2	60,680,616	60,680,762
C1ORF122	chr1	38,272,200	38,273,057
C7ORF50	chr7	1,083,209	1,084,163
CCDC144NL	chr17	20,798,895	20,799,770
CLIC1	chr6	31,698,492	31,699,299
DMPK	chr19	46,282,571	46,283,081
FAM86C1	chr11	71,498,202	71,499,118
FAM86JP	chr3	125,634,060	125,634,453
FOXK1	chr7	4,722,778	4,723,928
FSCN	chr7	5,635,134	5,635,954
GAPDH	chr12	6,641,602	6,642,355
GET4	chr7	914,964	915,832
GNB2	chr7	100,274,361	100,275,305
GPANK1	chr6	31,630,819	31,632,542
GPR45	chr2	105,857,809	105,859,084
KCNQ1	chr11	2,554,562	2,555,577
LDLRAD4	chr18	13,611,370	13,611,825
LMO3	chr12	16,760,040	16,761,003
LOC100133461	chr4	3,680,721	3,681,760
MIR22HG	chr17	1,617,363	1,618,296
MTMR8	chrX	63,614,857	63,615,496
N10	chr1	28,423,399	28,424,202
N12	chr5	3,593,413	3,594,276
N22	chr19	4,579,481	4,580,471
N23	chr14	106,004,434	106,004,608
N24	chr6	170,449,417	170,450,804
N27	chr6	30,432,200	30,433,944
N30	chr15	27,959,473	27,960,032
N8	chr11	69,260,136	69,261,045
N9	chr7	35,300,077	35,301,070
NCOR2	chr12	124,990,897	124,991,140
NONE	chr10	17,347,047	17,347,392
NSG1	chr4	4,386,726	4,387,698
PAX2	chr10	102,509,693	102,510,569
PITX1	chr5	134,365,728	134,366,535
PRSS22	chr16	2,908,157	2,908,935
PTPRN2.3	chr7	157,523,356	157,524,159
PTPRN2.4	chr7	158,109,339	158,110,153
PURA	chr5	139,492,535	139,493,491
PYY2	chr17	26,553,567	26,554,908
SECTM1	chr17	80,278,592	80,280,331
SEMA6B	chr19	4,555,999	4,556,983
SEZ6	chr17	27,330,794	27,332,647
SLC22A18AS	chr11	2,909,690	2,909,716
SOHLH1	chr9	138,590,204	138,590,996
THBS3	chr1	155,176,868	155,177,784
TNXB	chr6	32,064,146	32,065,891

### Technical validation / replicate performance

**Fig. 2:**
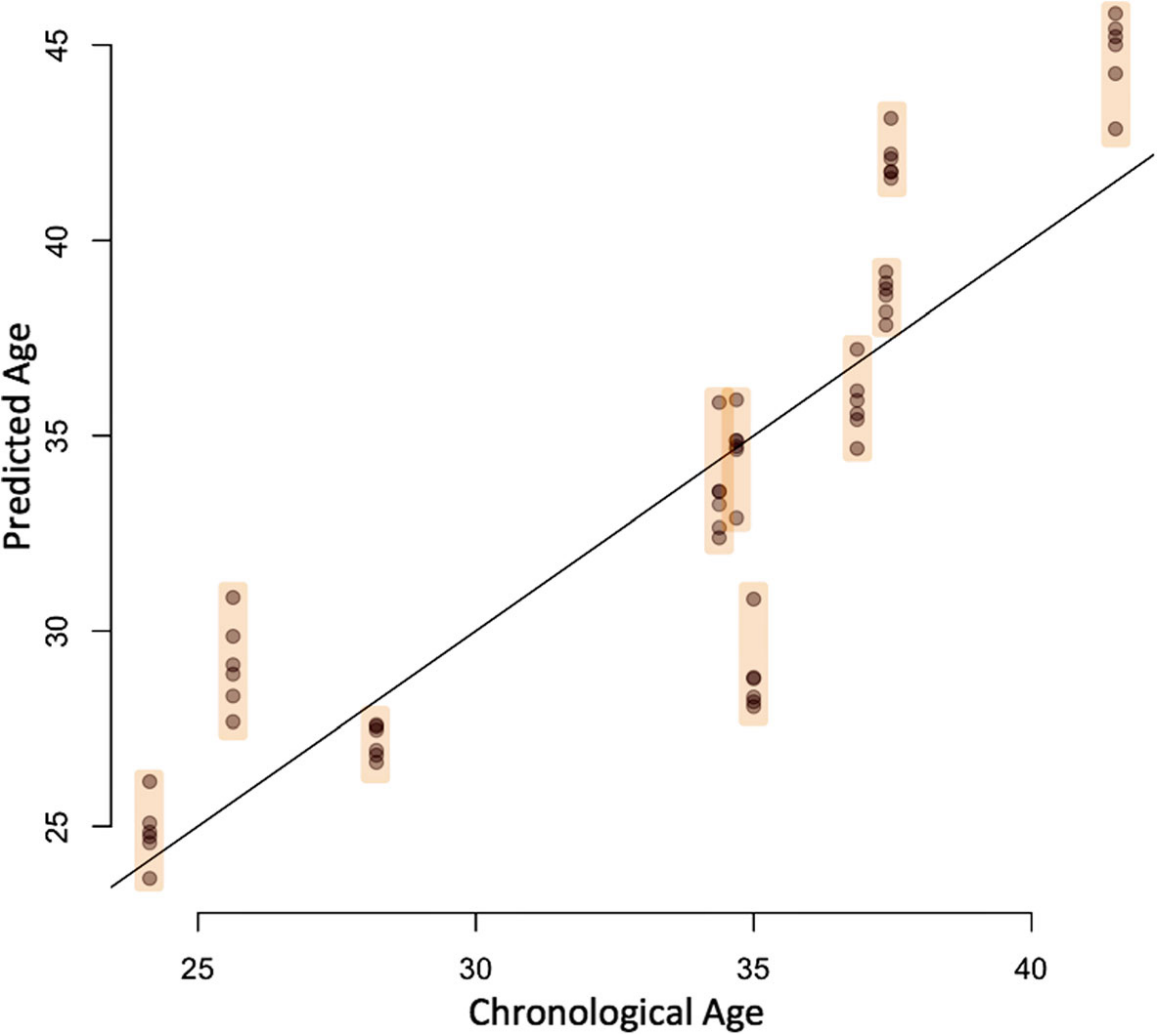
Scatterplot depicting the age prediction in a completely independent cohort of 10 samples each of which has 6 technical replicates. The points within each discrete orange box represent predictions for all six replicates from each individual. The line is representative of a “perfect” prediction

Because variability can be a concern in array experiments, we tested our model in a completely independent cohort of samples that were not used in any of our cross validation / model training experiments. We utilized 10 sperm samples, each with 6 replicates (a total of 60 samples) that were each run on the 450 K array platform from a previously published study [9]. Further, the samples from this study were exposed to varying extremes in temperature to test the stability of the sperm DNA methylation signatures. Thus these samples do not represent strict technical replicates (because of slight variations in treatment) but do provide an even more robust test of the algorithms predictive power on sperm DNA methylation signatures in multiple samples from the same individual. The model was applied to these samples and performed well in both precision and accuracy. Specifically, not only was the consistency of predictions in this independent cohort quite robust (SD = 0.877 years), but the accuracy of prediction was very similar to what was seen in the training data set with an MAE of 2.37 years (compared to 2.04 years in the training data set) and a MAPE of 7.05% (compared to 6.28% in our training data set). We additionally performed linear regression analysis on the predicted age vs. actual age in each of the 10 individuals in the dataset and found a significant association between these two (R^2^ of 0.766; *p* = 0.0016; Fig. 2).

### The impact of smoking on age prediction

**Fig. 3:**
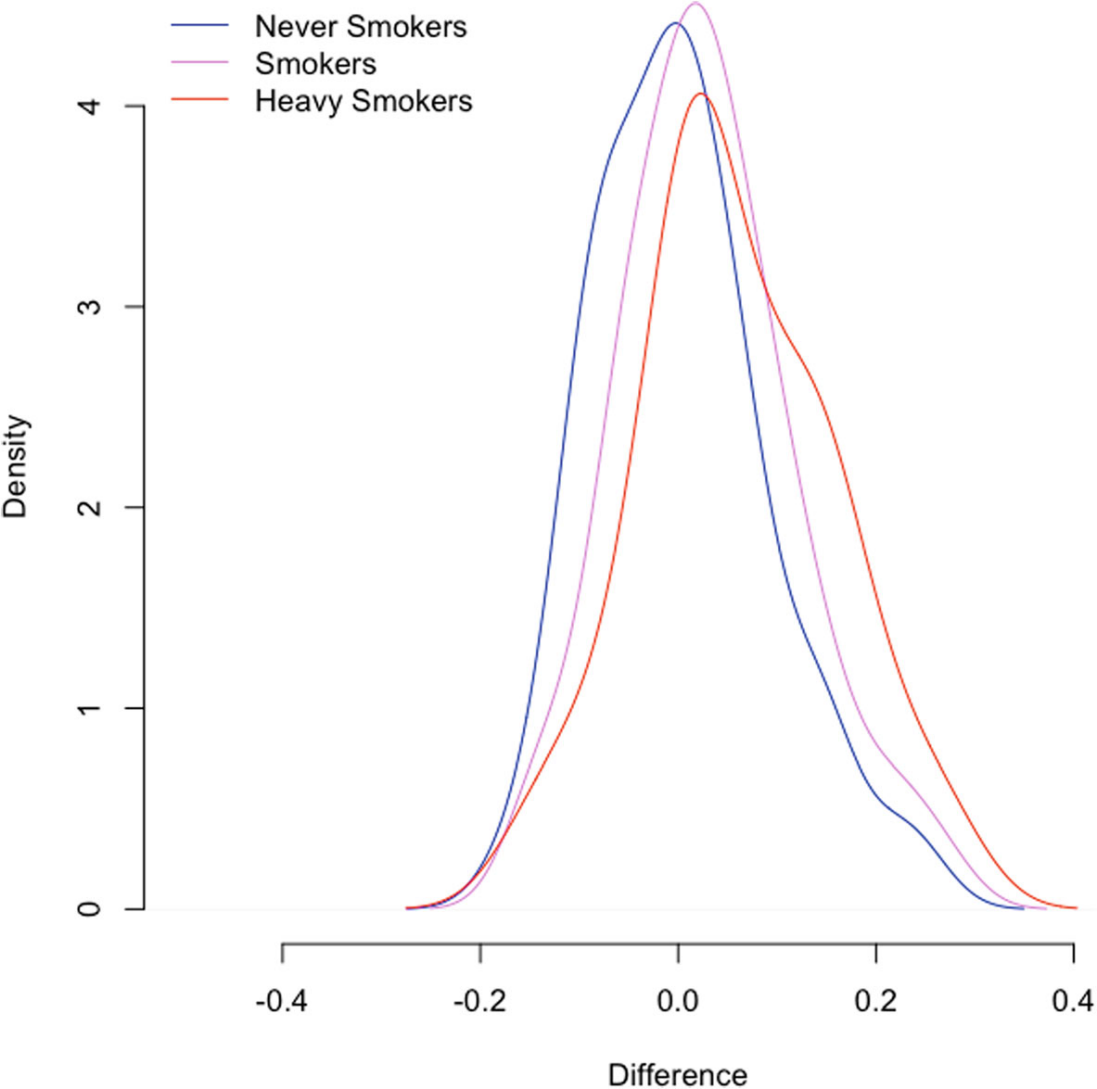
Density plot shows the accuracy of age prediction in never smokers, smokers, and heavy smokers among individuals below 35 years of age. Similar patterns exist in the entire cohort but are the most profound in this age group

To test the potential diagnostic/clinical utility of our model we have more closely assessed the data in our original cross validation dataset. Specifically we have analyzed our smoking dataset [7], which includes sperm methylation data from 78 smokers and 78 individuals who responded as “never smokers.” Similar aged men are represented in each group. We additionally isolated a portion of the smoking group who were had smoked cigarettes for > 10 years. We found an approximately 1.5% increased in predicted age compared to chronological age in all smokers and 2.5% increase in long term smokers. However this difference failed to reach statistical significance. Interestingly, this same pattern was observed (though significantly higher in magnitude) when screening only individuals who were less than 35 years old at the time of collection (Fig. 3). In these samples we saw a 3% increase in predicted age compared to chronological age in the smoker group and a nearly 6% increase in predicted age in the long-term smokers (*p* = 0.0196).

## Discussion

We have developed a sperm age calculator that has the capacity to identify an individual’s chronological age based only on their sperm DNA methylation signatures. Previous studies have defined aging patterns in somatic cells and one in particular [1] very successfully generated an aging calculator using methylation signatures of somatic cells. However, these findings do not hold true in sperm and further, the DNA methylation age calculator that described in 2013 fails to work effectively with paternal germ line epigenetic signatures. Herein, we have described the development of a linear model that has the ability to accurately predict ages with these signatures. Specifically our model is based on average methylation signatures at 51 genomic loci known to be altered as men age [2].

In the process of model construction, we evaluated multiple potential methods by which we could train our model. One important consideration was the nature of the population with which the model was trained. While there is a balance in selecting a population (broad applicability vs. targeted population) we decided to utilize a population with diverse fertility phenotypes and exposures to ensure that it could perform well with many different phenotypes. As such we included smokers and non-smokers, individuals of known fertility, those currently being treated for infertility, and men from our general population.

We also attempted to obtain a simple model with the fewest number of features needed to achieve optimal predicative power. Our initial approach was to focus only on the regions previously identified to be altered by age (based on previous data) and refined the model by only assessing these regions. The rational behind this approach stems from the understanding the changes to methylation signatures occurring in small regions of the genome (at promoters and CpG islands) have the potential to affect transcription and thus phenotype [10]. Thus, using fraction methylation across an entire region that has already been demonstrated to be associated with aging in sperm offers an approach that is likely the most directly tied to biological function. In fact, our own previous studies assessing these specific aging marks suggest that they may have biologic significance in the offspring of older males [2]. We compared models trained with this restricted feature set to models built using all available data (the entire array) as features. While the models built with the entire array did have increased predictive power when testing them in the same samples that were used for model training, there was no difference seen when predicting ages in a test group (to which the model was blinded during construction) between models built using the restricted and regionalized feature set and the whole array feature set. Importantly, the most efficient single model (considering only data from tests using samples that had not been used in training) was constructed using the restricted and regionalized feature set. The fact that models built using the entire array had improved performance in the training set without any improvement in the test set supported our approach to use a simplified and more restricted set of features for training. This is because when using the entire array, model construction was able to identify some features that appeared to be predictive of age but these were, in reality, only effective predictors in the samples used in training and thus added no benefit when training in samples that had not been used in model construction. We found that even in our model using regional level features there was some amount of simplification that could be performed. Indeed, we were able to scale our list of features from 148 regions down to 51 regions with the same predictive power. This effort resulted in a quite robust model with strong predictive power (an average of ~ 93% accuracy in predicting each individual’s age and an r^2^ of ~ 0.89).

Our data indicate that the model constructed herein is also technically robust. We were able to assess previous data from our lab in which 10 individuals had six technical replicates on 450 k methylation arrays [9]. This replicate data enabled to assess the power of the model in two distinct ways. First, we were able to assess the predictive power of the model on a completely independent cohort (each of these samples were assessed at a different time and in different batches of arrays than those upon which the model was trained). Second, we were able to show that the model is able to generate consistent predictions for individuals between technical replicates. Of additional interest is the fact that the samples used in these technical replicates originated from a study that tested the impact of extreme and prolonged temperature exposures on sperm DNA methylation patterns. Thus a portion of the replicates screened were exposed to various magnitudes of less than ideal conditions. However, it is important to note that we did observe a drop in r^2^ in our independent cohort. This is not an entirely unexpected finding due to the variation that can occur between different batches of arrays. In brief, we found that the array batch effects are sufficiently strong to slightly decrease the predictive power of the age calculator for batches performed outside of our original training set. In contrast, the model is sufficiently strong to overcome such variation with strong predictive power, though that power is slightly reduced compared to what is seen in our training/test data set. The ability to maintain predictive power, even when assessing other batches of data is important in a model that will have broad applicability. In the future as more data become available, the model can be updated with increased sample size and with additional batches of experiments, which will lead to even more robust predictive power.

It is important to note that due to the difficulty in obtaining samples for very young individuals and older individuals (those outside of the typical age of paternity) our model is constructed mainly using samples in men between 20 and 45 years of age. As a result, we expect that moving forward when samples from older men become available that the model should be updated to include more samples from men of different ages to improve the predictive power at other ages.

Our data also suggest that there may be some utility for such a model in a clinical setting. Specifically, we were able to identify an age-affect of smoking in our cohort of patients. We found that individuals who smoke appeared to have acceleration in the pattern of aging and thus the individual’s germ line age was in some cases significantly higher than their chronological age. However, this finding specifically should be taken in the proper context, where we only identified significant age acceleration in a portion of the data set and that (though all age ranges followed the same trend) the magnitude of the effect varied greatly. Still, this represents one example of many different analyses that could be performed in a clinical setting should further exploration identify consistent and impactful age acceleration. With future studies we may find that different levels/types of infertility, obesity, or other environmental exposures may cause acceleration in the aging pattern seen in sperm. One of the biggest questions that remains if such associations exist is the potential impact of this age acceleration. Such a pattern could potentially result in increased risk to offspring health, as epidemiological data clearly shows increased incidence of neuropsychiatric disease in the offspring of older fathers [11–16]. This increase in risk may not mean that the altered methylation pattern itself causes these offspring abnormalities, but instead the methylation signatures of age are simply a good indicator of the overall state or age of the sperm. Likely of more immediate interest to clinicians is the fact that advanced paternal age is associated with a loss of fecundity and fertility. Specifically, it has been shown that men older than 45 years take ~ 5 times longer to achieve a pregnancy as men less than 25 years (when controlling for female age) [17]. A similar decrease in fecundity was identified in a large population study in 2000 which showed that (after adjusting for maternal age) men > 35 years of age had a 50% lower chance of achieving a pregnancy within 12 months of attempting conception than younger men [18]. Other studies have also shown decreased fertilizing potential in both IUI and IVF [19, 20]. While the magnitude of this effect remains controversial [21, 22], it is clear that advanced paternal age does play an important role in a couple’s fertility status and can clearly result in, at a minimum, a significantly increased time to pregnancy. For many couples, such potential barriers to achieving a pregnancy are essential to understand and discuss with their care providers. While none of these associations have been proven in this specific work, the potential clinical utility of the calculator is clear and warrants further investigation both in predicting an individual’s health/fertility as well as in the prediction of abnormalities in the offspring.

It is important to note that while the findings of alterations associated with age in the sperm epigenome are intriguing, the direct impact of these alterations is still in question. In fact, the actual impact of any sperm epigenetic alteration on the embryo or the offspring is difficult to predict due to massive reprograming events that take place in the early embryo and in the primordial germ cells. However, data do suggest that methylation marks in many sub-telomeric regions escape reprograming events and can be potentially be passed on to the offspring [23–27]. Intriguingly, our original sperm aging study showed that the majority of age-affected regions were located in these sub-telomeic regions as well [2]. Such a transmission of age-affects would be remarkable, but may offer a real potential explanation for at least a portion of the downstream impact of paternal age on offspring disease incidence and phenotype.

The data described herein are quite promising, though some limitations are clear. Foremost among them is our knowledge of downstream impacts as described above. This will require a great degree of effort to determine the nature of these effects and if risks to fertility or the offspring can be modified in any way by various treatments. Further, while the current model is very effective at predicting an individuals age and is quite robust technically, the alterations we are observing to predict age are subtle and thus small inefficiencies can result in an inability to detect meaningful changes. Despite this, because of the approach we have taken in designing a model based only on limited numbers of regions there is a potential to modify this model for use with different platforms that may offer increased resolution and consistency, for example targeted sequencing [28]. With such an approach, we may be able to improve an already robust predictive model by multiplex sequencing with extreme depth at only the 51 sites of interest. This could provide an even more economical and reliable predictive model. Taken together, the data that we have shown here are intriguing and warrant a great deal of further investigation and also have the potential to be improved with future iterations.

## Conclusions

Similar to what is seen in somatic cells, our data clearly indicate that sperm methylation signatures are altered over time and that these modifications can be used to predict an individual’s age. This enabled the development of a germ line age calculator that is presented in this work. This calculator offers tremendous potential in the basic sciences to address fundamental questions about aging, fertility, and potential impacts on embryogenesis and even offspring health. In addition, while much work is yet required, it appears that such calculations may have potential benefit in clinical fertility care as well as in forensics. While the future potential is evident, the promise of the technology and its biological underpinnings will not be fully realized without significant future efforts in both animal models and in direct human studies.

## Methods

### Samples, study design, data availability

**Fig. 4:**
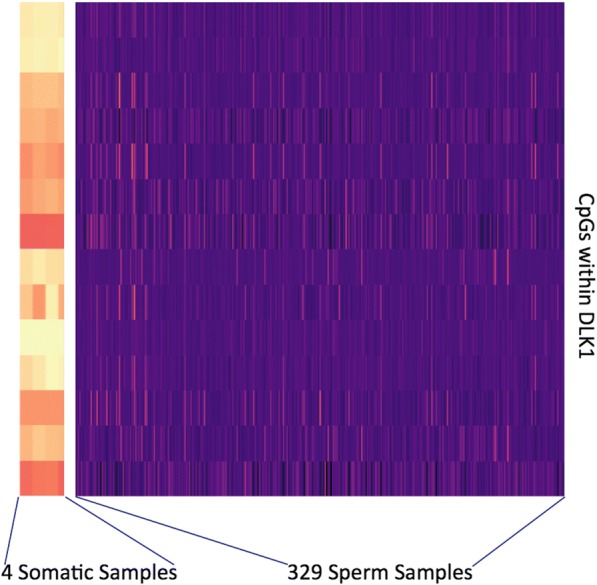
Heatmap of the DLK1 locus, which is highly differentially methylated between sperm and somatic cells is used to confirm the absence of contaminating signals in our data set. 4 blood samples are listed at the far left of the heatmap and the remainder of the samples used in our study follow

In the current study we assessed sperm DNA methylation array data from 3 distinct previously performed studies [2, 6, 7]. All of the studies were performed in our laboratory. We included only the samples for which ages were available. From these data sets, we were able to acquire a total of 329 samples that were used to generate the predictive model outlined herein. Each sample was run on the Illumina 450 K methylation array. In each case, we used SWAN normalization to generate beta-values (values between 0 and 1 that represent the fraction of a given CpG that is methylated) that were used in our study. During early processing of the sperm samples, great care was taken to ensure that no somatic cell contamination was present that could potentially influence the results of our studies. To confirm the absence of somatic cell contamination we assessed the methylation signatures at a number of sites throughout the genome, each of which are highly differentially methylated between sperm and somatic tissues. In Fig. 4, we show the differential methylation at one representative genomic locus, DLK1, to illustrate the absence of contaminating signals in the samples used in our study. While variability exists between the methylation in these samples there exists very little, if any somatic DNA methylation signals.

### Samples used

Individuals with many different fertility phenotypes provided the samples used in this study. Our training data set includes samples from sperm donors, known fertile individuals, infertility patients (including those seeking intrauterine insemination or even in vitro fertilization treatment at our facility), and individuals from the general population. Further, our data set includes those that have very different lifestyles and environmental exposures (heavy smokers and never smokers, Obese individuals and those with normal BMIs, etc.).

The average ages in each study were statistically similar (with averages of approximately 33 years of age) aside from the smallest study used [2], which previously assessed aging patterns (average age of approximately 44 years of age). Known fertile sperm donors collected ~ 27% of all samples used in the study. Individuals from the general population in the Salt Lake City area collected 31% of the samples and infertility patients collected another 42% of the samples used in the study. Of all the individuals included in our study approximately 26% are smokers. In terms of BMI, 46% of the men in our study were considered normal, 35% were considered overweight, and 9% were classified as obese.

### Model training

We utilized the glmnet package in R to facilitate training and development of our linear regression age prediction model [8]. For training of our model, we first tested multiple designs to generate the most robust and easily interpretable model. We first constructed a model trained on all CpGs on the entire array (“entire array” training). We additionally limited the training dataset to only 148 regions that we have previously identified to be strongly associated with the aging process to ensure the broad interpretability to the results of the model [2]. We trained two models within those 148 genomic regions to identify the best possible outcomes. First, we trained on all of the beta-values for each CpG located in our regions of interest (“CpG level” training). Second, we generated a mean of beta-values for each region that included the CpGs within each region respectively yielding mean beta-values for each region (“regional level” training), and the model was trained only on these averages.

In both of the above-described scenarios, we employed a 10-fold cross validation strategy to repeatedly test trainings on 90% of our samples and hold out 10% for a test set. This was performed 10 times on unique subgroups of the entire data set. The results from these ten validations were compared between the entire array training, the CpG level training, and the regional level training. To compare the accuracy and predictive power of these models we performed linear regression for each (actual age vs. predicted age) and generated r^2^ values. These r^2^ values were compared via simple two-tailed t-test to determine if any significant difference exists between the two approaches to model construction (CpG level construction vs. regional level construction).

### Technical validation / replicate performance

We tested our model in a completely independent cohort of samples [9]. We used 10 sperm samples each with six technical replicates that were each run on the 450 K array (not those used in our cross validation / model training) to determine the precision and consistency of prediction. These samples were all taken from men who were attending the Andrology lab for a fertility workup. In each case the men who provided the sample had normal semen analysis measures. Linear regression analysis of predicted vs. actual age was performed using R.

### The impact of smoking on age prediction

We tested 78 never smokers and 78 smokers using our age prediction model. Similar aged men are represented in each group. We additionally isolated a portion of the smoking group who have smoked cigarettes for > 10 years. In this analysis we compared accuracy of the age prediction of each group to determine if there is a significant increase in the age prediction compared to chronological age in individuals who smoke. We identified the percent difference between chronological age and predicted age and compared this value between smokers and non-smokers via two-tailed t-test to identify the presence of age acceleration.
